# AI-Assisted Surface-Enhanced Raman Spectroscopy for Cardiovascular Diagnostics: From Plasmonic Materials to Clinical Translation

**DOI:** 10.3390/nano16130785

**Published:** 2026-06-23

**Authors:** Anju Joshi, Gymama Slaughter

**Affiliations:** 1 Center for Bioelectronics, Old Dominion University, Norfolk, VA 23508, USA; 2 Department of Electrical and Computer Engineering, Old Dominion University, Norfolk, VA 23508, USA

**Keywords:** surface-enhanced Raman spectroscopy, artificial intelligence, cardiovascular disease, cardiac biomarkers

## Abstract

Raman spectroscopy (SERS) has emerged as a powerful analytical technique, offering molecular fingerprint specificity and ultrasensitive detection of cardiac biomarkers. Recent advances in plasmonic nanostructures, surface functionalization strategies, and flexible sensing platforms have significantly improved the analytical performance of SERS-based biosensors. In parallel, the integration of artificial intelligence (AI) and machine learning has enabled robust interpretation of complex spectral datasets, facilitating automated biomarker classification and improved diagnostic accuracy in heterogeneous biological environments. Despite these advances, the field remains fragmented, with limited integration between nanomaterial design, biomarker selection, and data-driven analysis, and persistent challenges related to reproducibility, standardization, and clinical validation. This review provides a comprehensive and critical synthesis of AI-assisted SERS platforms for cardiovascular diagnostics, integrating advances in plasmonic materials, biomolecular recognition, and intelligent spectral analysis within a unified framework. It further examines key translational barriers, including data variability, model interpretability, and scalability, and outlines future directions for developing standardized, edge-deployable, and clinically validated SERS-AI systems.

## 1. Introduction

Cardiovascular diseases (CVDs) remain the leading cause of mortality worldwide, accounting for approximately 17.9 million deaths annually [1,2]. According to the 2026 American Heart Association Heart Disease and Stroke Statistics Report, nearly half of all U.S. adults aged ≥20 years are affected by CVD, corresponding to an estimated prevalence of 49% (approximately 130.6 million individuals) [3]. In addition to the substantial clinical burden, CVDs impose significant economic challenges, as healthcare expenditures are projected to rise from $193.1 billion to $414 billion [4,5]. Major behavioral risk factors contributing to cardiovascular diseases include unhealthy dietary habits, physical inactivity, tobacco use, and excessive alcohol consumption [6]. Therefore, the early identification of at-risk individuals, combined with timely therapeutic intervention and continuous disease monitoring, is essential to reduce the escalating clinical, societal, and economic burden associated with CVDs.

Conventional clinical assessment of cardiac function primarily relies on centralized imaging modalities, which are often limited by insufficient molecular specificity, high operational costs, and potential radiation-related risks [7]. These imaging approaches are frequently complemented with blood-based biomarker assays, including brain natriuretic peptide (BNP), N-terminal pro-BNP (NT-proBNP), cardiac troponins (cTnI and cTnT), and electrolytes (Na^+^ and K^+^) to identify biochemical and molecular abnormalities associated with cardiac dysfunction [8,9]. Conventional cardiac biomarker assays are often limited by delayed biomarker elevation following myocardial injury, inadequate sensitivity for early-stage disease detection, poor specificity in complex inflammatory conditions, and dependence on centralized laboratory infrastructure [10,11]. Moreover, existing blood-based assays are not readily adaptable for rapid multiplexed analysis, continuous monitoring, or integration into portable and wearable diagnostic systems [12,13].

Raman spectroscopy has gained considerable attention owing to its label-free, non-destructive, and radiation-free molecular detection capability [14]. The technique generates unique vibrational fingerprints of biomolecules without the need for fluorescent labels or contrast agents, making it highly attractive in complex biological matrices [15]. Conventional Raman spectroscopy suffers from low Raman scattering cross-sections and therefore, SERS has emerged as a highly sensitive analytical platform capable of dramatically amplifying Raman signals through plasmonic nanostructures. Here, an interaction between incident light and noble metal nanostructures, such as gold (Au) and silver (Ag) nanoparticles, leads to localized surface plasmon resonance (LSPR) and creates intense electromagnetic “hotspots” to enable near single-molecule-level detection sensitivity [16]. Despite its remarkable analytical sensitivity, interpreting SERS spectra remains highly challenging due to spectral complexity, signal variability, substrate heterogeneity, molecular orientation effects, and overlapping vibrational signatures [17]. Conventional spectral analysis methods, including baseline correction, peak assignment, and linear multivariate analysis, seem incompetent to accurately interpret highly heterogeneous and nonlinear spectral datasets generated from complex biological samples [18]. To tackle the associated complexities, various artificial intelligence (AI) and machine learning (ML) approaches have been explored for efficient analysis of complex, high-dimensional SERS datasets. The integration of AI/ML with SERS has significantly improved automated spectral interpretation, biomarker classification, disease prediction, and real-time cardiovascular monitoring, while overcoming limitations of conventional data analysis approaches. Such integration has the potential to transform cardiovascular healthcare through precise, automated, rapid, and cost-effective diagnostic solutions.

There have been several recent reviews highlighting the importance of integrating AI and ML to process and analyze complex SERS data, including spectral classification, nanostructure optimization, and diverse biosensing applications [19,20,21]. These review articles are largely technology-centric across broad biomedical and analytical applications and do not clearly indicate the application of AI/ML for SERS-based sensing of cardiac biomarkers to predict the early onset of cardiovascular diseases. Therefore, this review focuses on cardiac biomarker selection, SERS strategies, advanced plasmonic nanostructure engineering, biomolecular recognition strategies, and AI/ML-enabled spectral intelligence within a unified framework for cardiovascular diagnostics. Also, it critically evaluates challenges in spectral variability, substrate engineering, data standardization, applicability of AI models, and clinical validation, while highlighting emerging opportunities in wearable and point-of-care cardiovascular monitoring, multimodal sensing platforms, and edge-deployable machine learning systems. By bridging technological innovation with clinical translation, this review provides a distinct and timely framework for accelerating the development and adoption of intelligent SERS platforms in precision cardiovascular healthcare.

## 2. Biomarkers Relevant to Cardiovascular Disease

**Figure 1:**
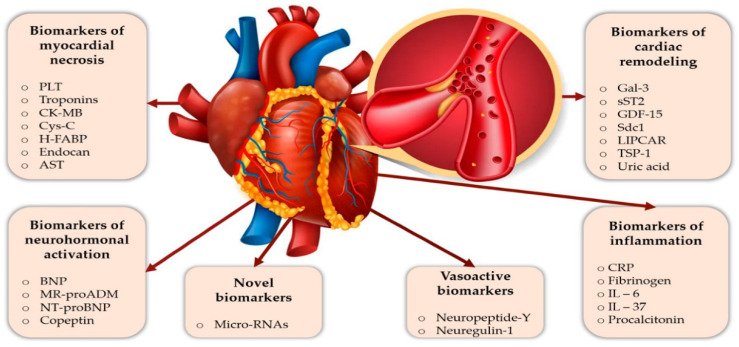
Different biomarkers associated with cardiovascular disease. Adapted with permission from [22].

Cardiovascular diseases, including arrhythmia, hypertension, atherosclerosis, myocardial infarction, and heart failure, are highly complex disorders involving multiple interconnected molecular pathways. A wide range of biomolecules, including proteins, peptides, enzymes, hormones, metabolites, and microRNAs (miRNAs), have emerged as important cardiovascular biomarkers [13,22] (Figure 1). Based on their underlying pathophysiological roles, cardiovascular biomarkers can be broadly categorized into several major classes, as discussed in the subsequent sections.

### 2.1. Cardiac Injury Biomarkers

Cardiac injury biomarkers are molecules released into circulation following myocardial damage. Their concentrations depend on the extent of cardiac injury, as they are rapidly released after injury and remain detectable within a clinically relevant diagnostic window, enabling timely diagnosis and risk assessment while minimizing interference from recurrent injury, particularly in acute myocardial infarction (AMI) [23]. Among these, cardiac troponins (cTnI and cTnT) are regarded as the gold standard for myocardial injury, while creatine kinase-MB (CK-MB) and myoglobin serve as complementary biomarkers that provide additional insight into myocardial necrosis, temporal kinetics, and risk stratification. Therefore, a convenient and scalable approach was proposed using magnetic molecularly imprinted polymers employing a characteristic peptide as a template and MAA and DMAm as functional monomers. Here, an engineered plasmonic substrate was developed using a ZrO_2_ shell layer wrapped around Fe_3_O_4_ magnetic cores modified using silver nanostructures [24]. These fabricated SERS substrates were further modified using 4-MBA via Ag-SH bonds to produce Raman signals, attributed to the shielding effect. In another report, an antibody-oriented engineering strategy was explored to enable sensitive detection of cTnT using Raman reporter (IR-808) functionalized gold nanoparticles as SERS nanotags, where the resonance match between IR-808 and a 785 nm excitation laser provides enhanced signal amplification to the detection process, leading to the rapid target enrichment reduce background interference, and highly sensitive cTnT detection within 7 min [25]. Myoglobin (Mb), an early biomarker of AMI, has been detected using label-free SERS platforms based on engineered plasmonic nanostructures [26]. The efforts were further extended by using a three-dimensional silver nanopinetree array (Ag NPT/ITO), which generated abundant electromagnetic hotspots, enabling Mb detection in buffer and urine samples with a detection limit of 10 ng/mL [27]. These studies demonstrate the potential of plasmonic SERS substrates for sensitive detection of early cardiac injury biomarkers.

### 2.2. Heart Failure Biomarkers

Brain natriuretic peptide (BNP) is a key biomarker for heart failure (HF) and is widely used for diagnosis, prognosis, and disease monitoring. BNP is secreted in response to ventricular pressure overload and wall stress, and its levels correlate with disease severity and clinical outcomes [28,29]. Consequently, sensitive SERS-based platforms have been developed for BNP detection to support early diagnosis and effective HF management. A fiber-optic microfluidic SERS-based immunosensor was reported for the detection of BNP utilizing MOF-functionalized Au-HEPES nanoparticles labeled with toluidine blue as SERS nanotags, achieving a detection limit of pg/ML with low sample consumption and high throughput, demonstrating its potential for point-of-care CHF monitoring [30].

Additionally, galectin-3 (Gal-3), a macrophage-derived β-galactosidase-binding protein, is implicated in cardiac fibrosis, inflammation, and ventricular remodeling [31]. By promoting fibroblast activation and collagen deposition, Gal-3 contributes to HF progression. Elevated levels correlate with increased mortality and poor outcomes in both HFpEF and HFrEF populations. However, its incremental diagnostic value over natriuretic peptides remains debated, limiting its role primarily to fibrosis-related risk stratification rather than standalone diagnosis [32]. Soluble suppression of tumorigenicity-2 (sST2) is an emerging biomarker for heart failure (HF) and myocardial infarction, with elevated levels associated with inflammation, fibrosis, adverse cardiac remodeling, and poor clinical outcomes. As a decoy receptor for interleukin-33 (IL-33), sST2 disrupts cardioprotective signaling, and concentrations above 35 ng/mL are strongly linked to increased mortality and rehospitalization risk [33,34]. Furthermore, its relative independence from age, obesity, and renal dysfunction enhances its value for prognostic assessment and patient management.

### 2.3. Inflammatory Biomarkers

Inflammation plays a central role in the development and progression of cardiovascular diseases, making inflammatory biomarkers valuable tools for risk assessment and prognosis. C-reactive protein (CRP), serum amyloid A (SAA), and interleukin-6 (IL-6) are among the most extensively studied markers. A SERS-based lateral flow assay using AuMBA@mSiO_2_ nanotags enabled simultaneous detection of SAA and CRP with detection limits of 0.1 ng/mL and 0.05 ng/mL, respectively, while supporting smartphone-assisted point-of-care analysis [35]. Similarly, functionalized gold nanoparticles enabled sensitive IL-6 detection over the range of 0.002–1 ng/mL, with a detection limit of 0.00091 ng/mL [36]. In addition, a laser-induced graphene (LIG)-based immunosensor has been reported for sensitive electrochemical detection of CRP, demonstrating high analytical performance and compatibility with scalable, flexible point-of-care diagnostic platforms [37].

### 2.4. Genetic and Epigenetic Biomarkers

Cell-free nucleic acids (cfNAs), including cell-free DNA (cfDNA) and circulating mitochondrial DNA (cf-mtDNA), have emerged as promising cardiovascular biomarkers because they reflect real-time cellular injury, inflammation, and tissue remodeling. In myocardial infarction, cfDNA is rapidly released into circulation within hours of symptom onset, with levels correlating with infarct size, troponin concentration, and clinical outcomes, highlighting its potential for early diagnosis, risk stratification, and monitoring of myocardial injury [38,39,40]. In parallel, microRNAs (miRNAs) provide critical epigenetic insights into cardiovascular pathology [41]. Sensitive detection of miR-21, a microRNA implicated in cardiac remodeling and fibrosis, has been demonstrated using a paper-based biosensor, enabling low-cost and portable nucleic acid quantification [40]. Similarly, a disposable biosensor was developed for the quantitative detection of miRNA-141, further expanding the toolkit for point-of-care miRNA biosensing [42].

A dual-mode paper-based microfluidic platform integrating colorimetric and SERS detection was developed for the analysis of miR-29a, a microRNA biomarker associated with myocardial infarction [43]. The colorimetric component enabled rapid visual screening and discrimination of miR-29a concentrations ranging from 18–360 ng/mL, while the SERS modality provided more sensitive and reproducible semi-quantitative analysis. Owing to its enhanced analytical performance, the SERS assay achieved a detection limit of 47 ng/mL. By combining simple visual detection with sensitive spectroscopic confirmation, this platform demonstrates significant potential for point-of-care cardiovascular biomarker monitoring and early disease diagnosis. Currently, no single biomarker provides sufficient specificity, sensitivity, and temporal resolution for reliable clinical decision-making. As a result, current strategies increasingly rely on multimarker panels that integrate biochemical, inflammatory, and genetic signals. However, challenges related to variability, standardization, and real-time measurement persist, particularly for continuous or wearable monitoring systems, emphasizing the need for integrated, multiplexed, and context-aware diagnostic frameworks.

## 3. SERS-Based Detection of Cardiac Biomarkers: Strategies, Applications, and Limitations

Raman spectroscopy is considered a promising analytical technique for molecule identification, characterization of molecular interactions, and monitoring dynamic changes during chemical reactions. However, the performance of Raman spectroscopy is limited by the inherently weak Raman scattering cross-section of molecules, resulting in low signal intensity. In 1974, Martin Fleischmann and coworkers reported a remarkable enhancement in the Raman signal of the pyridine molecule adsorbed on roughened silver electrodes, marking the discovery of SERS [44]. Since then, the field has experienced tremendous growth over the past five decades, and SERS is now recognized as a powerful analytical platform with promising applications in disease diagnostics, food safety, environmental monitoring, and biomedical sensing.

### 3.1. SERS-Based Sensing Mechanism

**Figure 2:**
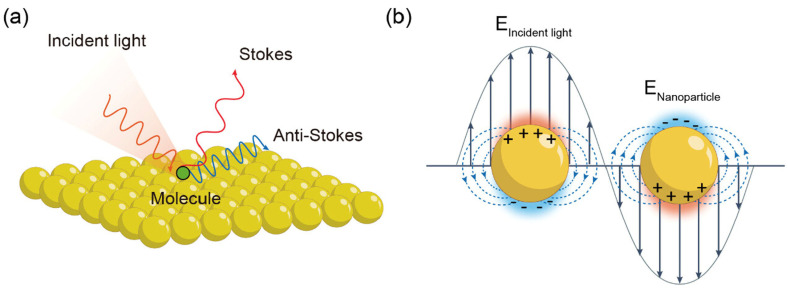
Detailed representation of the surface-enhanced Raman scattering of (**a**) a molecule adsorbed on gold nanostructure undergoing Stokes and anti-Stokes Raman scattering and (**b**) the generation of localized surface plasmon resonance contributing to SERS. Adapted with permission from [14].

There are two main mechanisms (electromagnetic (EM) and chemical enhancement) for the SERS effect. The EM enhancement mechanism is considered the dominant contributor to SERS signal amplification. This effect occurs in substrates that generate LSPR upon light excitation, leading to the formation of highly intensified local electromagnetic fields, or “hotspots,” that significantly amplify Raman scattering signals (Figure 2). The location of the plasmon resonance (*ω*) is considered as [45]:

(1) $$\omega =\sqrt{(4\pi Ne^{2}}\notin m*)$$

Here *N* is the free carrier density and m∗ is the effective mass of the charge carriers (electrons or holes). Based on this relationship, noble metals such as copper (Cu), Ag, and Au, which possess high free electron densities (10^22^–10^23^ cm^−3^), exhibit plasmon resonance in the visible region and are therefore widely used as efficient plasmonic SERS substrates. In addition, degenerate semiconductors with tunable free-carrier densities (~10^16^–10^21^ cm^−3^) can exhibit metal-like plasmonic behavior and have recently emerged as promising alternative SERS substrates due to their adjustable optical and electronic properties [15]. The EM enhancement mechanism is generally independent of the chemical nature of the analyte molecules and is primarily governed by the intensity of localized electromagnetic fields generated near plasmonic nanostructures. These fields can be tuned by modulating the nanostructure morphology, dielectric properties, and interparticle plasmonic coupling, thereby forming highly intensified electromagnetic “hot spots” between neighboring metallic nanoparticles [46,47]. However, the non-uniform distribution of nanoparticles in terms of size and shape, along with their tendency to aggregate, results in randomly generated hot spots with inconsistent field enhancement. Therefore, undesirable variations in SERS signals are often observed, limiting reproducibility. To overcome these challenges, advanced plasmonic nanostructures with controlled, reproducible hot spots have been developed using both bottom–up and top–down fabrication methods, including shell-isolated nanoparticle dimers with tunable nanogaps and lithographically fabricated ordered nanoparticle arrays [48,49]. Although, the EM mechanism successfully explains the strong enhancement of Raman scattering through LSPR-induced amplification of the local electromagnetic field, different enhancement magnitudes observed among various vibrational modes cannot be fully understood by EM theory. To address these limitations, a chemical mechanism (CM) was proposed, attributing SERS enhancement to charge-transfer (CT) interactions between the substrate and the adsorbed molecule [50]. These interactions modify the molecular electron density distribution, increase molecular polarizability, and consequently enhance Raman scattering. Typically, the CM contributes to an enhancement factor of approximately 10^2^–10^3^ in metal–molecule systems.

According to Jensen and co-workers, three major CT-related processes, interfacial ground-state charge transfer (GSCT), photoinduced charge transfer (PICT), and molecular resonance Raman scattering (RRS), contribute to the chemical enhancement mechanism [51]. GSCT is a non-resonant process involving chemisorption interactions between the molecule and the substrate in the ground state, leading to redistribution of electron density and modification of the molecule’s intrinsic Raman cross-section. In contrast, PICT is a wavelength-dependent resonant process in which electrons are transferred between the substrate and molecule upon laser excitation. Depending on the relative alignment of the metal Fermi level and the molecular HOMO/LUMO energy levels, CT can occur either from the metal to the molecule or vice versa, with maximum enhancement occurring when the CT transition involves energy levels close to the Fermi level [52].

The third process, RRS, occurs when the excitation laser energy matches the intrinsic electronic transition energy of the molecule, resulting in substantial Raman signal enhancement ranging from 10^2^ to 10^6^ [53]. The enhanced local electromagnetic fields produced by plasmonic nanostructures dramatically improve Raman scattering sensitivity, enabling the detection of low-abundance analytes with high specificity [54]. EM and CM highlight a fundamental trade-off in SERS-based sensing. While EM enhancement provides extremely high sensitivity through localized plasmonic “hotspots,” it is highly dependent on nanoscale geometry and often results in poor reproducibility across substrates. In contrast, CM offers improved molecular specificity through CT interactions but contributes relatively lower signal amplification. As a result, achieving both high sensitivity and reproducibility remains a key challenge in SERS-based detection systems. These limitations are further amplified in complex biological environments, where variability in analyte distribution and surface interactions can significantly impact signal stability. Furthermore, the development of SERS platforms for real-world applications requires precise control over nanostructure design, improved standardization, and integration with data-driven approaches to ensure reliable quantitative analysis. Moreover, SERS-based biosensing strategies can be broadly categorized into label-based and label-free approaches, each offering distinct advantages and limitations in terms of sensitivity, complexity, reproducibility, and clinical applicability [55].

### 3.2. Label-Based SERS Detection of Cardiac Biomarkers

Label-based SERS detection relies on the indirect identification of target biomarkers through Raman reporter molecules conjugated to plasmonic nanostructures. A typical SERS nanotag consists of four key components: (i) a metallic nanostructure (e.g., Au or Ag) for signal enhancement, (ii) a protective shell to improve stability and biocompatibility, (iii) a Raman reporter molecule to generate a unique spectral fingerprint, and (iv) a target-specific recognition element such as antibodies or aptamers. This approach enables exceptionally high sensitivity and multiplexing capability, as distinct Raman reporters allow simultaneous detection of multiple biomarkers. For example, Zheng et al. developed a near-infrared SERS-based immunoassay for BNP detection using a fiber-integrated microfluidic platform, achieving pg/mL level sensitivity with rapid analysis [30]. The study investigated a SERS probe developed using metal–organic framework-functionalized Au-HEPES coupling nanoparticles (AuNPs) conjugated to the toluidine blue (TB) dye (Raman reporter). The signal intensity was further enhanced using a SERS-active substrate embedded at the bottom of the microfluidic channel when the SERS-tagged BNP complexes flowed through the device. In another report, a magnetic-assisted multiplex SERS biosensor employing Au@Ag nanotags and Fe_3_O_4_-based capture substrates enabled simultaneous ultrasensitive detection of multiple HF biomarkers, namely, cTnI, NT-proBNP, and sST2 at fg/mL detection limits, highlighting its strong potential for multiplexed clinical diagnostics of heart failure biomarkers [56]. The target-specific aptamers and antibodies were conjugated onto the nanoparticle surfaces to construct a sandwich-type Fe_3_O_4_–Ag/HF biomarker/Au@Ag composite structure for selective target recognition. Recent advances have further expanded label-based SERS platforms by leveraging engineered nanostructures and integrated systems featuring core–shell architectures (e.g., Au@Ag nanogaps), metasurfaces, and hierarchical plasmonic substrates to maximize electromagnetic field enhancement.

### 3.3. Label-Free SERS Detection

Label-free SERS detection represents a simpler alternative in which analyte molecules are directly detected based on their intrinsic Raman signatures without the use of reporter molecules. This approach eliminates the need for complex nanotag fabrication and enables more straightforward sensor design. Recent developments have focused on molecularly imprinted polymers (MIPs) and surface-functionalized substrates to enhance selectivity. For example, Xu et al. developed an epitope-oriented molecularly imprinted polymer (hg-EMIP)-based SERS biosensor for cTnI detection, utilizing host–guest interactions between β-cyclodextrin and ferrocene for efficient template immobilization, followed by the synthesis of a multifunctional imprinted polymer layer for precise template immobilization and selective recognition [57]. Unlike conventional reporter-assisted SERS systems, the developed assay enabled direct Raman detection of cTnI without the use of extrinsic nanotags. The platform demonstrated high specificity, broad detection range, and successful validation in human serum samples. While label-free SERS offers a simplified detection strategy with reduced probe complexity, achieving reliable, sensitive, and reproducible measurements in clinical settings remains a significant challenge.

Label-based and label-free SERS strategies illustrate a fundamental trade-off between sensitivity, complexity, and reproducibility. Label-based platforms offer high sensitivity and multiplexing capability through engineered nanotags and signal amplification but often involve complex fabrication and limited scalability. In contrast, label-free approaches offer simpler assay formats and direct molecular interrogation but often yield weaker signals and greater susceptibility to interference from complex biological matrices. Despite remarkable advances, a key challenge remains translating highly sensitive SERS measurements into reliable, quantitative, and clinically actionable information. Variability in nanostructure fabrication, hotspot distribution, surface chemistry, and sample heterogeneity continue to limit reproducibility and standardization. Moreover, the strong emphasis on achieving ultralow detection limits often overshadows equally important factors such as quantitative accuracy, robustness, and performance under clinically relevant conditions.

### 3.4. Integrated SERS Applications for Cardiac Biomarker Detection

Extensive efforts have been devoted to engineering nanostructured SERS substrates with enhanced surface area and plasmonic activity to maximize receptor immobilization and improve analytical sensitivity. Among the various nanomaterials explored, gold nanostructures have emerged as attractive sensing platforms owing to their excellent biocompatibility, chemical stability, and compatibility with well-established surface functionalization strategies, particularly thiol–gold chemistry for biomolecular immobilization. In this regard, Mabbott et al. introduced a microfluidic paper-based analytical device (μPAD) integrated with colorimetry transduction method for sensitive detection of miR-29a, a critical biomarker for CVD [43]. Here, the authors synthesized gold nanoparticles via citrate reduction and further functionalized them with the Raman reporter malachite green isothiocyanate (MGITC) to produce a Raman readout. The assay achieved a limit of detection (LoD) of 47 ng/mL, demonstrating the feasibility of integrating plasmonic nanostructures with low-cost paper-based microfluidic platforms for nucleic acid sensing. Such point-of-care biosensing platforms are increasingly being coupled with digital connectivity tools, including smartphone-assisted readout, cloud-based data management, and wireless transmission to enable real-time and remotely accessible cardiovascular diagnostics [58].

**Figure 3:**
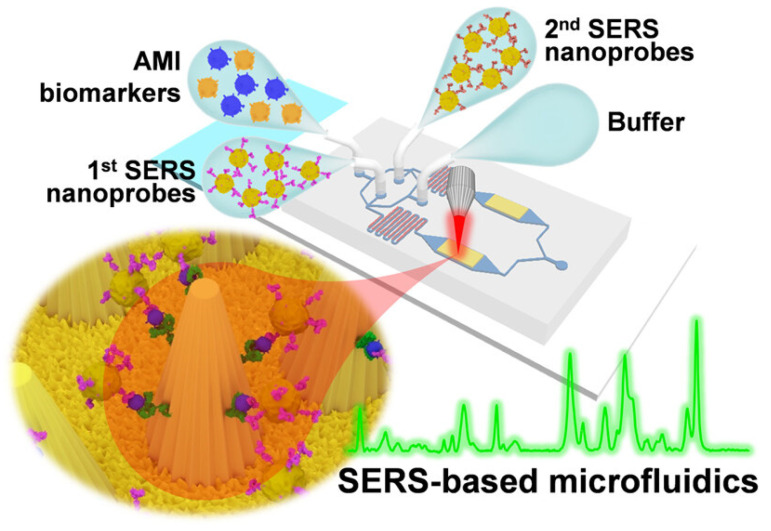
Schematic representation of the SERS-Based Immunoassay on a microfluidic chip with plasmonic microcones for the detection of myocardial infarction biomarkers. Adapted with permission from [58].

To further enhance analytical performance, nanostructured microfluidic platforms incorporating hierarchical plasmonic architectures have been developed. A plasmonic nanostripe microcone array (PNMA)-embedded microfluidic chip was reported for the simultaneous detection of creatine kinase-MB (CK-MB) and cardiac troponin I (cTnI) (Figure 3) [59]. Unlike conventional microtiter-plate immunoassays, the device incorporated parallel microfluidic channels integrated with high-surface-area, gold-coated, hierarchical nano–microstructures, enabling efficient antibody immobilization and improved analyte capture. This architecture achieved a LoD of 0.1 ng/mL for both biomarkers. The substantial improvement in sensitivity was attributed to the increased receptor loading capacity and enhanced electromagnetic field localization provided by the hierarchical nanostructures. The role of advanced plasmonic engineering was further demonstrated by Zheng et al., who developed pyramidal plasmonic metasurfaces composed of alternately stacked Au–SiO_2_ meta-atoms [60]. Unlike conventional SERS sensors that rely solely on intensity variations, this platform employed nanomechanical perturbation-induced Raman frequency shifts as the sensing mechanism. The metasurface generated spatially extended electric and magnetic field-enhanced hotspots, enabling highly sensitive and reproducible detection of CK-MB, myoglobin (Mb), and cTnI. The resulting LoDs were 0.05 ng/mL, 3.8 ng/mL, and 0.007 ng/mL for CK-MB, Mb, and cTnI, respectively. In addition to ultralow detection limits, the platform demonstrated multiplexing capability, highlighting the advantages of combining engineered plasmonic architectures with novel SERS transduction strategies.

Nanoparticle morphology has also been shown to significantly influence SERS performance. Xiang et al. developed a sandwich-type SERS immunosensor employing multi-tipped gold nanostars (AuNSs) as both substrates and nanotags for cTnI detection [61]. The sharp protrusions of the AuNSs generated intense localized electromagnetic fields and enhanced plasmonic coupling between the immunosubstrate and nanotags. Consequently, the AuNS-based platform produced nearly threefold greater Raman enhancement than conventional gold nanospheres, achieving an LoD of 0.00909 ng/mL^−1^ with excellent reproducibility. These findings underscore the importance of anisotropic nanostructure design for maximizing hotspot density and signal amplification. Beyond gold nanostructures, hierarchical silver-based architectures have also been investigated. A three-dimensional silver nanopinetree array (Ag NPT/ITO) was developed for SERS detection of myoglobin, exploiting the abundance of electromagnetic hotspots generated within its multiscale architecture [27]. The platform achieved an LoD of 10 ng/mL, illustrating the capability of three-dimensional plasmonic structures to improve analyte accessibility and Raman signal enhancement.

Although gold and silver nanostructures individually exhibit excellent plasmonic properties, hybrid nanostructures combining multiple metallic components have been explored to further enhance sensing performance. Similarly, Wu et al. developed an ultrasensitive multiplex SERS biosensor that combined Au@Ag nanoparticles as SERS nanotags with Ag-coated Fe_3_O_4_ magnetic nanoparticles serving as capture substrates. The Au@Ag nanotags were encoded with Raman reporter molecules and functionalized with antibodies or aptamers specific to target biomarkers, while the Fe_3_O_4_–Ag particles enabled magnetic enrichment and efficient target capture [56]. The resulting sandwich complexes significantly enhanced immunorecognition efficiency and signal amplification, achieving LoDs of 0.000001 ng/mL for cTnI, 0.0001 ng/mL for NT-proBNP, and 0.000001 ng/mL for soluble suppression of tumorigenicity 2 (sST2). Importantly, validation using 45 clinical serum samples demonstrated strong agreement with conventional clinical assays, highlighting the platform’s translational potential.

In addition to advances in nanomaterial engineering, the integration of chemometric and machine-learning approaches has significantly enhanced the quantitative capabilities of SERS biosensors. Lim et al. developed a calibration-free SERS-based μPAD for the simultaneous quantification of glycogen phosphorylase isoenzyme BB (GPBB), CK-MB, and cardiac troponin T (cTnT) for early diagnosis of AMI [62]. The platform used three antibody-conjugated SERS nanotags, each encoding a distinct Raman reporter, enabling multiplex biomarker identification via unique spectral fingerprints. To eliminate dependence on conventional calibration curves, partial least squares (PLS) predictive modeling was incorporated for direct quantification of unknown serum samples. The device achieved LoDs of 0.008 ng/mL for GPBB, 0.01 ng/mL for CK-MB, and 0.001 ng/mL for cTnT, all substantially below clinically relevant thresholds. By combining ultrasensitive multiplex detection with calibration-free quantitative analysis, this platform demonstrates considerable promise for point-of-care AMI screening and improved clinical decision-making.

Additionally, substrate engineering approaches aimed at increasing hotspot density, signal uniformity, and analytical reproducibility are gaining considerable attention. For example, Yoo et al. developed a heat-treated nickel foam substrate coated with gold nanostructures to generate a three-dimensional hotspot-rich SERS-active surface for competitive immunoassay-based detection of cTnI [63]. The porous architecture of the nickel foam provided a large effective surface area for biomolecule immobilization while promoting the formation of numerous plasmonic hotspots. Such three-dimensional substrates offer advantages over conventional planar SERS platforms by enhancing analyte accessibility and reducing signal variability. However, maintaining uniform gold deposition throughout the complex porous structure and ensuring batch-to-batch reproducibility remain important challenges that may affect large-scale manufacturing and clinical translation.

Moving beyond substrate engineering alone, recent studies have increasingly integrated advanced molecular amplification strategies with SERS transduction to achieve unprecedented analytical sensitivity. Li et al. developed an ultrasensitive dual-mode electrochemiluminescence (ECL)/SERS biosensor for B-type natriuretic peptide (BNP) detection by combining split-aptamer recognition, T7 transcription, CRISPR/Cas13a-mediated signal amplification, and interfacial toehold-mediated strand displacement reaction (TSDR) on a CsPbBr_3_@PDA@Au-modified electrode [64]. In this design, target recognition activated a T7–Cas13a amplification cascade that removed ferrocene/Raman co-labeled probes, producing a turn-on ECL signal and a corresponding turn-off SERS signal for ratiometric quantification. The synergistic integration of multiple amplification mechanisms enabled BNP detection at attomolar concentrations while maintaining excellent selectivity against structurally related biomarkers, including NT-proBNP, and robust performance in complex biological matrices.

Although such amplification-assisted platforms represent a significant advance in analytical sensitivity, they also highlight an emerging trend in SERS biosensing where increasingly sophisticated signal amplification strategies are used to compensate for intrinsic limitations in target abundance. While attomolar-level detection demonstrates the remarkable capability of combining CRISPR-based amplification with plasmonic sensing, the practical clinical value of achieving such extreme sensitivity warrants careful consideration, as BNP concentrations in clinical samples are typically several orders of magnitude higher than the reported detection limits. The continued evolution of SERS platforms toward wearable and portable configurations represents a critical translational frontier, as evidenced by recent advances in wearable biosensors designed for continuous, non-invasive monitoring of chronic disease-relevant biomarkers in biofluids such as sweat and interstitial fluid [65]. Collectively, these studies demonstrate that rational engineering of nanostructured SERS substrates, including hierarchical architectures, plasmonic metasurfaces, magnetic–plasmonic hybrids, and core–shell nano assemblies, has dramatically improved the sensitivity, multiplexing capability, and clinical applicability of SERS-based cardiovascular biomarker detection. The progression from simple gold nanoparticle substrates to sophisticated multifunctional plasmonic nanostructures highlights the central role of hotspot engineering, enhanced biomolecular immobilization, and signal amplification in achieving ultrasensitive cardiovascular diagnostics.

## 4. AI/ML-Assisted SERS for Cardiac Biomarker Detection: Workflow, Algorithms, and Clinical Applications

The integration of AI and ML with SERS has emerged as a powerful strategy to address key limitations associated with spectral complexity, biological variability, and substrate heterogeneity. While advances in plasmonic nanostructures have significantly improved signal enhancement, interpreting high-dimensional SERS spectra remains a major challenge due to overlapping vibrational signatures, background interference, and experimental variability. AI/ML-assisted frameworks provide automated, data-driven approaches for spectral analysis, enabling improved classification accuracy, biomarker identification, and diagnostic decision-making in cardiovascular diseases. AI-assisted SERS analysis typically follows a workflow comprising spectral acquisition, preprocessing, feature extraction, dimensionality reduction, model training, validation, and prediction [66]. Raman spectra acquired from biological samples such as serum, plasma, saliva, or extracellular vesicles often contain noise arising from fluorescence background, instrumental artifacts, and substrate variability. Consequently, preprocessing steps, including baseline correction, denoising, smoothing, normalization, and cosmic-ray removal, are essential for improving spectral quality and consistency. Feature extraction and dimensionality reduction techniques, such as principal component analysis (PCA), linear discriminant analysis (LDA), t-distributed stochastic neighbor embedding (t-SNE), and uniform manifold approximation and projection (UMAP), are then employed to reduce data complexity while preserving diagnostically relevant information [67,68]. Subsequently, supervised or unsupervised ML algorithms are used for disease classification, biomarker quantification, and pattern recognition. Model performance is commonly assessed using metrics such as accuracy, sensitivity, specificity, precision, F1-score, and AUC-ROC, often coupled with cross-validation to improve robustness [69]. However, the reliability of AI-assisted SERS analysis remains highly dependent on experimental consistency, as variations in sample preparation, substrate fabrication, spectral acquisition, and preprocessing can introduce biases that affect model performance.

**Figure 4:**
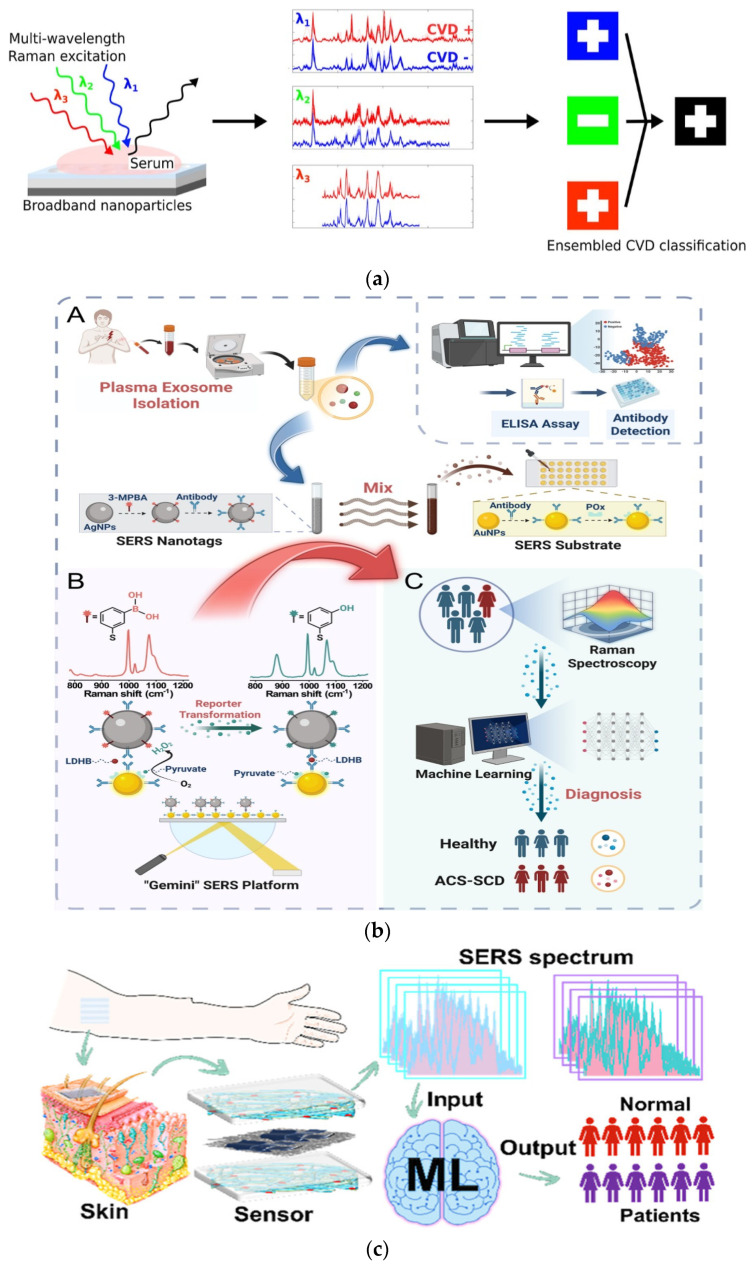
Application of AI-SERS-based sensors for detection of (**a**) cTnI, BNP, CRP, creatinine, LDL; (**b**) Pyruvate and Lactate Dehydrogenase: (**A**) multiomics-guided biomarker discovery; (**B**) dual-analyte SERS sensing mechanism; (**C**) SVM-based exosome classification. (**c**) Cholesterol. Adapted with permission from [70,72,75].

Dixon et al. developed a SERS-AI platform capable of simultaneously detecting multiple cardiovascular biomarkers, including cTnI, BNP, CRP, creatinine, and low-density lipoprotein (LDL), in simulated serum samples [70]. Here, two different ML algorithms (RF and PCA-SVM) were evaluated on 300 serum samples for the label-free classification of clinically relevant biomarker concentrations (Figure 4a). The investigated algorithms effectively reduced spectral dimensionality, extracted diagnostically relevant features, and differentiated physiological and pathological biomarker profiles. PCA-based methods have shown strong capability in distinguishing AMI from healthy controls by identifying dominant spectral variations associated with disease states. PCA machine learning model maximizes overall variance rather than class-specific variance, limiting sensitivity for subtle biomarker changes. Random Forest models are well-designed to mitigate this limitation by capturing complex nonlinear relationships within high-dimensional spectral datasets and providing improved robustness against noise and overfitting. However, both ML models rely predominantly on experimental conditions (laser excitation wavelength, SERS substrate morphology, sample preparation protocols, and spectral preprocessing methods), limiting model transferability across different datasets and clinical settings. An interesting case study by Liu and coworkers recently demonstrated that integrating machine learning with SERS significantly enhanced the analytical utility of BNP sensing by extracting diagnostically relevant information from highly complex, noisy spectral datasets [71]. Here, five different ML algorithms, including decision trees, random forests, SVM, KNN, and voting classifiers, were employed to classify BNP-associated Raman spectra obtained from antibody-functionalized nanofinger substrates. Here, KNN outperforms other ML algorithms with 100% classification accuracy on the test dataset. These findings demonstrate the potential of ML to uncover subtle spectral patterns that are difficult to identify through conventional peak-based analysis or visual inspection. However, the reported performance was derived from a relatively small cohort comprising serum samples from only 9 patients and 9 healthy controls, suggesting model overfitting and generalizability across diverse patient populations. Although multiple Raman spectra were collected from each sample, spectral replication does not fully compensate for limited biological variability, and the reported accuracies may overestimate real-world performance. Moreover, the study relied on a highly engineered nanofinger substrate capable of generating exceptionally strong and reproducible SERS enhancement, making it challenging to isolate the true contribution of the ML algorithms from that of the underlying sensing platform. Therefore, further efforts should focus not only on classification accuracy but also on robust quantitative prediction of BNP concentrations across clinically relevant ranges.

In another report, gemini-based SERS dual-mode systems were developed for the simultaneous detection of pyruvate dehydrogenase (proteins) and pyruvate (metabolites) from plasma-derived exosomes, as shown in Figure 4b [72]. Here, AuNPs were functionalized with an LDHB-specific antibody and a boronate-based SERS nanotag responsive to a hydrogen peroxide-mediated cascade. The assay achieved detection limits of 2.415 μM for pyruvate and 0.032 ng/mL for LDHB at a single platform. Here, the application of SVM classifier achieved 85% accuracy (90% sensitivity, 80% specificity) in distinguishing ACS-SCD patients from healthy controls, comparable to conventional methods (AUC = 0.82 vs. 0.79). Naz et al. demonstrated the utility of both Principal Component Analysis (PCA) and Partial Least Squares Regression (PLSR) for analyzing SERS spectra of serum samples from patients with AMI and varying cTnI levels [73]. PCA effectively reduced spectral dimensionality and differentiated healthy individuals from AMI patients by identifying disease-associated spectral features. However, as an unsupervised method, PCA does not directly relate spectral variations to biomarker concentrations. In contrast, PLSR enabled quantitative prediction of cTnI levels by modeling the relationship between SERS spectra and analyte concentration, achieving R^2^ values of 0.92 and 0.87 for calibration and prediction, respectively. These findings highlight PCA as a useful tool for spectral discrimination and PLSR for quantitative biomarker assessment in SERS-based diagnostics.

MXene-based two-dimensional nanomaterials have recently emerged as a compelling class of functional materials for wearable biosensing, owing to their exceptional electrical conductivity, large active surface area, and chemically tunable surface terminations; recent design frameworks have highlighted their potential for integration into autonomous, real-time biosensing architectures operating across a range of biofluids [74]. Wearable SERS platforms using flexible AgNW/MXene composite substrates are gaining attention for cholesterol detection in sweat, achieving stable performance under repeated mechanical deformation [75]. The high specific surface area of MXene and AgNWs enhanced the adsorption of target cholesterol molecules, enabling cholesterol detection at 10^−8^ M over 50 continuous stretch–release cycles (Figure 4c). Three ML algorithms, including latent Dirichlet allocation (LDA), RF, and XGBoost, were implemented to build models that can accurately distinguish an unhealthy person from a healthy individual. The RF achieved the best performance, with an accuracy of 83.5% and AUC of 0.924, outperforming LDA and XGBoost. The integration of ML models such as RF and XGBoost enabled the classification of healthy versus unhealthy individuals, demonstrating the potential of combining wearable sensing with AI-driven analytics.

A detailed performance analysis of various ML models indicated that the current application of ML for analyzing complex SERS datasets lacks large, diverse clinical datasets, limiting model generalizability (Table 1). Most studies rely on small, proof-of-concept cohorts, increasing the risk of overfitting and reducing robustness in real-world settings. Furthermore, variations in sample preparation, instrumentation, and spectral acquisition can introduce batch effects and spectral drift, compromising model reliability. The limited interpretability of many AI/ML models, particularly deep learning approaches, also remains a major challenge, as their “black box” nature hinders clinical trust and regulatory acceptance.

Collectively, these findings indicate that no single machine-learning algorithm is universally optimal for SERS-based cardiovascular diagnostics; instead, algorithm selection should be tailored to the characteristics of the spectral dataset and the required balance between predictive performance, robustness, and interpretability. Although ML models have demonstrated promising diagnostic accuracy, many studies rely on relatively small cohorts, increasing the risk of overfitting and limiting generalizability. Reports of exceptionally high classification accuracies (>95%) should therefore be interpreted cautiously, as they may reflect dataset-specific biases or potential data leakage. Furthermore, the limited interpretability of advanced AI models remains a barrier to clinical adoption. Future efforts should focus on explainable AI frameworks, standardized spectral preprocessing and data-acquisition protocols, external validation across diverse patient populations, and uncertainty quantification to improve the reliability and translational potential of AI-assisted SERS diagnostics.

**Table 1:** Performance analysis of different ML models towards the SERS-based sensing of cardiac biomarkers.

ML Model	Cardiovascular Target	SERS Platform	Task Type	Performance Metric	Interpretability	Strengths	Limitations	Representative Study
PCA	cTnI	AgNP-based SERS	Feature extraction	Clear discrimination of AMI and healthy serum spectra	High	Identifies dominant spectral variations and enables visualization of disease-related clustering.	Primarily an exploratory dimensionality-reduction tool and not a standalone predictive model.	[73]
PLSR	cTnI	AgNP-based SERS	Regression	RMSEC = 2.98 ng mL^−1^; RMSEP = 3.98 ng mL^−1^; R^2^cal = 0.92; R^2^pred = 0.87	High	Demonstrated an accurate correlation between spectral data and cTnI concentrations.	Requires calibration with known biomarker concentrations.	[73]
PCA-SVM	cTnI, BNP, CRP, Creatinine, LDL	AgNP-based SERS	Classification	Accuracy = 99 ± 3.2%	Moderate	Successfully distinguished physiological and pathological biomarker mixtures using label-free multiwavelength SERS spectra with reduced dimensionality and computational cost.	Sensitive to variations in SERS substrate morphology and experimental conditions.	[70]
Random Forest (RF)	cTnI, BNP, CRP, Creatinine, LDL	AgNP-based SERS	Classification	Accuracy = 98.0 ± 4.2%	Moderate	Suitable for high-dimensional SERS datasets and small sample sizes.	Lower accuracy than PCA-SVM in this study.	[70]
Decision Tree (DT)/Random Forest (RF)	BNP (early AMI warning)	PMMA/Au collapsible nanofinger SERS substrate	Classification	DT: 94.0%, CV = 89.81%; RF: 95.0%, CV = 93.58%	High	DT provided interpretable decision rules, while RF improved classification accuracy and generalization performance.	DT is susceptible to overfitting in high-dimensional Raman datasets; RF reduces but does not completely eliminate this issue.	[71]
Support Vector Machine (SVM)	BNP	PMMA/Au collapsible nanofinger SERS substrate	Classification	Accuracy = 96.3%	Moderate	Effective for high-dimensional nonlinear Raman spectra and achieved 96.3% accuracy.	Requires extensive parameter tuning and optimization.	[71]
K-Nearest Neighbors (KNN)	BNP	PMMA/Au collapsible nanofinger SERS substrate	Classification	Accuracy = 100%; 10-fold CV = 95.09%	Moderate–High	Achieved 100% prediction accuracy; simple implementation with only one hyperparameter (k).	Requires storage of the entire training dataset and can become computationally intensive for large datasets.	[71]
Voting Classifier	BNP	PMMA/Au collapsible nanofinger SERS substrate	Classification	Accuracy = 98.11%; 10-fold CV = 94.70%	Low–Moderate	Maintained high performance by combining complementary strengths of DT, RF, KNN, and SVM.	Performance depends on the quality and diversity of constituent classifiers.	[71]
LDA	Cholesterol/CVD sweat signatures	AgNW/MXene wearable SERS sensor	Classification	AUC = 0.91; Accuracy > 80%	High	Efficient dimensionality reduction and discrimination of complex sweat SERS spectra.	Lower diagnostic performance than RF.	[75]
XGBoost	Cholesterol/CVD sweat signatures	AgNW/MXene wearable SERS sensor	Classification	AUC = 0.904; Accuracy > 80%	Moderate	Efficient gradient-boosting model capable of handling sparse data and reducing overfitting.	Inferior performance to RF in this dataset.	[75]
Random Forest (RF)	Cholesterol/CVD sweat signatures	AgNW/MXene wearable SERS sensor	Classification	AUC = 0.924; Accuracy = 83.5%	Moderate	Best overall classification performance among evaluated models, achieving AUC = 0.924 and accuracy = 83.5% for non-invasive CVD detection from sweat SERS spectra.	Relatively small cohort (13 healthy controls and 25 CVD patients); larger validation studies are needed.	[75]

## 5. Conclusions and Future Perspectives

Cardiovascular diseases remain a leading cause of global morbidity and mortality, emphasizing the urgent need for sensitive, reliable, and real-time diagnostic technologies capable of enabling early detection and continuous monitoring. In this context, SERS has emerged as a powerful analytical tool, offering exceptional sensitivity, molecular specificity, and multiplexing capability for the detection of cardiac biomarkers. Recent advances in plasmonic nanostructures, including nanoparticles, hierarchical architectures, and meta-surfaces, have significantly enhanced signal amplification, enabling ultra-low detection limits and improved analytical performance. The growing exploration of emerging biomarkers, such as exosome-derived nucleic acids and inflammatory mediators, further expands SERS’s potential for early-stage diagnosis and personalized cardiovascular care. Additionally, identifying biomarkers in non-invasive biofluids, such as sweat and saliva, offers a transformative opportunity for continuous, patient-friendly monitoring. Importantly, many SERS-based platforms remain confined to laboratory-scale demonstrations and have limited validation in large, clinically diverse cohorts. Despite these advancements, several challenges hinder the clinical translation of SERS-based biosensing in complex biological environments. Furthermore, the lack of standardized protocols for data acquisition, preprocessing, and performance evaluation significantly affects reproducibility and cross-study comparability. The integration of AI and ML with SERS has demonstrated considerable promise in addressing spectral complexity and enabling robust biomarker classification. However, current AI frameworks often rely on cloud-based processing, limiting their suitability for real-time, point-of-care applications and introducing latency, data privacy concerns, and dependence on external computational infrastructure. The development of explainable, lightweight, and edge-deployable models will be essential for translating SERS-AI systems into portable diagnostic platforms. Future research must prioritize hardware-software co-optimization, focusing on the development of reproducible SERS substrates integrated with embedded AI for on-device spectral analysis. Moreover, multimodal data integration combining SERS with electrochemical sensing and imaging can provide a more comprehensive understanding of disease progression and enhance predictive accuracy. In addition, practical considerations such as cost, scalability, and infrastructure requirements must be addressed to ensure broader accessibility, particularly in resource-limited settings. Ethical challenges, including data privacy, algorithmic bias, and regulatory compliance, must also be carefully managed to enable safe and equitable implementation. Ultimately, while the convergence of SERS, advanced nanomaterials, and artificial intelligence represents a transformative paradigm for cardiovascular diagnostics, its clinical translation will depend on rigorous standardization, large-scale validation, and the ability to deliver robust, interpretable, and scalable solutions in real-world healthcare environments.

## Data Availability

No new data were created or analyzed in this study. Data sharing is not applicable to this article.
